# Tumour angiogenesis and tumour cell proliferation as prognostic indicators in gastric carcinoma.

**DOI:** 10.1038/bjc.1995.331

**Published:** 1995-08

**Authors:** K. Maeda, Y. S. Chung, S. Takatsuka, Y. Ogawa, N. Onoda, T. Sawada, Y. Kato, A. Nitta, Y. Arimoto, Y. Kondo

**Affiliations:** First Department of Surgery, Osaka City University Medical School, Japan.

## Abstract

**Images:**


British Journal of Cancer (1995) 72, 319-323
© 1995 Stockton Press All rights reserved 0007-0920/95 $12.00

### Tumour angiogenesis and tumour cell proliferation as prognostic indicators in gastric carcinoma

K Maeda, Y-S Chung, S Takatsuka, Y Ogawa, N Onoda, T Sawada, Y Kato, A Nitta, Y Arimoto, Y Kondo and M Sowa

*First Department of Surgery, Osaka City University Medical School, Japan.*

**Summary** Tumour growth depends on neovascularisation and tumour cell proliferation. Factor VIII-related antigen (F-VIII RA) localises to vascular endothelium. Expression of proliferating cell nuclear antigen (PCNA) is correlated with cell proliferation. We investigated the correlation between the expression of these antigens and prognosis in gastric carcinoma. A total of 108 specimens resected from patients with gastric carcinoma were investigated by staining with monoclonal antibodies against F-VIII RA and PCNA. Microvessel count (MVC; the mean number of microvessels in the five areas of highest vascular density at 200 × magnification) and PCNA labelling index (PCNA LI; percentage of positive cells in more than 500 tumour cells) were determined. The results showed that prognosis was significantly worse in patients who had a tumour with a high MVC (16 or greater) or a high PCNA LI (42% or greater) than in those patients who had a tumour with a low MVC (less than 16) or a low PCNA LI (less than 42%). Furthermore, MVC was significantly associated with the risk of hepatic recurrence. In conclusion, both MVC and PCNA LI may be good prognostic indicators in patients with gastric carcinoma.

**Keywords:** angiogenesis; cell proliferation; prognosis; gastric carcinoma

Recently, it has been suggested that the degree of tumour angiogenesis is related to malignant potential (Folkman, 1990; Bosari *et al.*, 1992). Cellular proliferative activity has also been suggested as a useful marker for the malignant potential of various carcinomas (Sowa *et al.*, 1988; Van Dierendonck *et al.*, 1989). In recent years, many studies have reported on the relationship between tumour angiogenesis, tumour cell proliferation and clinical outcome (Bouzubar *et al.*, 1989; Weidner *et al.*, 1992; Gasparini *et al.*, 1994). Weidner *et al.* (1992) have shown that, in patients with breast cancer, both relapse-free and overall survival rates decrease with increasing microvessel count. In this study microvessels were highlighted by staining for factor VIII-related antigen (F-VIII RA) or von Willebrand factor, which can be localised to vascular endothelium in tissue sections by immunofluorescence and immunoperoxidase techniques. Proliferating cell nuclear antigen (PCNA), an auxiliary protein for DNA polymerase $\delta$, plays an important role in DNA synthesis and is thought to be localised to nuclei, particularly during late $G_1$ and S-phases (Garcia *et al.*, 1989; Landberg *et al.*, 1990). In this context, PCNA has drawn attention as a marker for cell proliferation.

In this study, we investigated the correlation between prognosis and angiogenesis and cell proliferation in gastric cancer as demonstrated by immunohistochemical staining of F-VIII RA and PCNA respectively.

### Materials and methods

### *Clinical material*

Resected specimens from 108 patients with gastric carcinoma who underwent curative surgical resection at our institution were studied. The patients ranged in age from 28 to 78 years (mean age 59.3 years); 90 were men and 18 were women. No patient had received chemotherapy or radiation therapy before the surgery. The General Rules for Gastric Cancer (Japanese Research Society for Gastric Cancer, 1981) were used for the pathological diagnosis and classification of variables. In this study, tumours were divided into two histological subgroups: differentiated type, which consisted of papillary and tubular adenocarcinomas; and undifferentiated type, which consisted of poorly differentiated adenocarcinomas, signet ring cell carcinomas and mucinous adenocarcinomas. All patients were followed up for at least 5 years after surgery and routinely studied by diagnostic imaging (computerised tomography or ultrasonography) once or twice a year. The type of recurrence was established by diagnostic imaging, serum level of tumour marker, cytology, biopsy or surgery, and was classified as hepatic recurrence, peritoneal recurrence or another type of recurrence. Almost all patients had only one site of recurrence. In patients who had multiple sites of recurrence, the type of recurrence was determined at the time of first relapse.

Specimens were fixed in 10% formaldehyde and embedded in paraffin. Four-micrometre-thick sections were cut and mounted on glass slides.

### *Antibodies and reagents*

As primary antibodies, mouse monoclonal antibodies F8/86 (which recognises F-VIII RA) and PC10 (which recognises PCNA) were used at a dilution of 1:200. These antibodies were purchased from Dakopatts (Glostrup, Denmark). Normal rabbit serum, normal mouse immunoglobulin G (IgG), biotinylated rabbit anti-mouse IgG, streptavidin–peroxidase reagent and diaminobenzidine were purchased from Nichirei Corporation (Tokyo, Japan).

### *Immunohistochemical techniques*

Immunohistochemical studies were performed by the streptavidin–biotin method. Sections were dewaxed in xylene, taken through ethanol and then incubated with 0.3% hydrogen peroxide in methanol for 30 min to block endogenous peroxidase activity. Sections were then washed in phosphate-buffered saline (PBS) and incubated in 10% normal rabbit serum for 20 min to reduce non-specific antibody binding. Specimens were then incubated with primary antibodies at room temperature for 2 h, followed by three washes with PBS. Sections were then incubated with biotinylated rabbit anti-mouse IgG at a dilution of 1:100 for 30 min followed by

Correspondence: K Maeda, First Department of Surgery, Osaka City University Medical School, 1-5-7 Asahimachi Abeno-ku, Osaka, 545, Japan
Received 11 November 1994; revised 14 March 1995; accepted 16 March 1995.

three washes. Slides were then treated with streptoavidin–peroxidase reagent for 30 min at a dilution of 1:100 and were washed with PBS three times. Finally, slides were incubated in PBS containing diaminobenzidine and 1% hydrogen peroxide for 10 min, counterstained with methyl green and mounted. Normal mouse IgG was substituted for primary antibody as the negative control. Slides were interpreted for antigen expression by two investigators without knowledge of the corresponding clinicopathological data. Individual tumours demonstrated considerable heterogeneity in microvessel density and the expression of PCNA. Therefore, in each case, several blocks of the same tumour were retrieved and sections were cut and stained by antibodies. Microvessel counting and PCNA scoring were determined among the five areas with the highest density of these antigens from all tissue blocks.

*Microvessel counting*

Any brown-staining endothelial cell or cluster of endothelial cells that was clearly separate from adjacent microvessels, tumour cells and other connective tissue elements was considered as a single vessel (Figure 1). Branching structures were counted as a single vessel unless there was a break in the continuity of the structure. The stained sections were screened at 5 × magnification to identify the areas of highest vascular density within the tumour from all tissue blocks. These high-vascularity areas could occur anywhere within the tumour, but occurred most frequently at the margins of the carcinoma. Sclerotic areas, where microvessels were sparse, and areas immediately adjacent to benign tissue were not considered in vessel counts. Vessels were counted in the five areas of highest vascular density at 200 × magnification (× 20 objective and × 10 ocular, 0.785 $mm^2$ per field). Microvessel count (MVC) was expressed as the mean number of vessels in these areas. The two investigators' counts were significantly correlated (by Spearman rank correlation test; $r = 0.621$, $P < 0.01$), therefore the average of the two investigators' counts was taken for further analysis.

*Scoring of PCNA*

Only nuclear staining was accepted as positive (Figure 2). All labelled nuclei were regarded as positive. Nuclei from more than 500 tumour cells were counted microscopically among the five areas with the highest density of PCNA expression from all tissue blocks. The PCNA labelling index (PCNA LI) was calculated as the percentage of positive cell nuclei. Although the two investigators did not agree exactly regarding the labelling index, a significant association was observed between these two values (by Spearman rank correlation test; $r = 0.609$, $P < 0.01$). So the average of these two values was used for the study.

*Statistical methods*

The relationship between MVC, PCNA LI and clinicopathological factors was examined by the Wilcoxon rank sum test. Survival curves were calculated using the Kaplan–Meier method and analysed by the log-rank test. The influence of each variable on survival was assessed by Cox's proportional hazard model (Cox, 1972). The relationship between MVC, PCNA LI, various clinicopathological factors and the mode of recurrence was examined by chi-square test or logistic regression analysis. Statistical significance was defined as $P < 0.05$.

### Results

*MVC and PCNA LI*

Microvessel count ranged from 5.1 to 50.0 with a mean value, plus or minus the standard deviation, of 15.9 ± 10.3. PCNA LI ranged from 7.7% to 76.5% with a mean value of

**Figure 1** Immunohistochemical staining for F-VIII RA in cancer tissues of the stomach. Expression of factor VIII-related antigen. Microvessels represented by brown staining of endothelium stand out sharply from stromal tissue in a differentiated gastric adenocarcinoma (original magnification × 200).

**Figure 2** Immunohistochemical staining for PCNA in cancer tissues of the stomach. Expression of proliferating cell nuclear antigen. Nuclear staining in a high percentage of tumour cells in a differentiated gastric adenocarcinoma (original magnification × 400).

41.8% ± 19.5%. There was a wide standard deviation in both MVC and PCNA LI. No significant association was observed between these two variables (by Spearman rank correlation test; $r = 0.196$, $P = 0.069$).

*Relationship between MVC, PCNA LI and clinicopathological factors*

Neither MVC nor PCNA LI was associated with patients' age and sex. Table I shows the correlation between MVC and various clinicopathological factors. There was no statistically significant association between MVC and histological type, growth pattern or depth of invasion. However, the microvessel count in patients with lymph node metastases was significantly higher ($P < 0.01$) than in those without lymph node metastases.

Table II shows the correlation between PCNA LI and various clinicopathological factors. Significant differences existed with respect to serosal invasion and lymph node metastasis.

*Association of MVC, PCNA LI and other factors with survival*

Among the 108 patients who underwent curative resection, 29 died of disease recurrence. To evaluate the association

**Table I** Correlation between clinicopathological factors and microvessel counts

| *Variable* | | *Microvessel counts* *Mean ± s.d.* | *(median, range)* | P-*value* |
|---|---|---|---|---|
| Serosal invasion | | | | |
| Negative | ($n$ = 66) | 16.3 ± 11.3 | (12.0, 3.0–35.5) | NS |
| Positive | ($n$ = 42) | 15.5 ± 9.5 | (13.5, 5.1–50.0) | |
| Histological type | | | | |
| Differentiated | ($n$ = 50) | 15.2 ± 10.4 | (13.4, 3.0–50.0) | NS |
| Undifferentiated | ($n$ = 58) | 16.9 ± 10.8 | (14.0, 4.3–45.0) | |
| Growth pattern | | | | |
| Expanding | ($n$ = 47) | 16.3 ± 11.8 | (12.4, 4.5–44.3) | NS |
| Infiltrative | ($n$ = 61) | 16.0 ± 9.8 | (14.4, 3.0–50.0) | |
| Lymph node metastasis | | | | |
| Negative | ($n$ = 59) | 13.3 ± 9.1 | (10.2, 4.3–44.3) | <0.01 |
| Positive | ($n$ = 49) | 18.7 ± 11.3 | (17.6, 3.0–50.0) | |

**Table II** Correlation between clinicopathological factors and PCNA labelling index

| *Variable* | | *PCNA labelling index* *Mean ± s.d.* | *(median, range)* | P-*value* |
|---|---|---|---|---|
| Serosal invasion | | | | |
| Negative | ($n$ = 66) | 35.5 ± 13.2 | (35.0, 7.7–60.5) | <0.05 |
| Positive | ($n$ = 42) | 48.5 ± 16.9 | (48.5, 10.0–76.5) | |
| Histological type | | | | |
| Differentiated | ($n$ = 50) | 38.4 ± 16.4 | (38.9, 7.7–74.2) | NS |
| Undifferentiated | ($n$ = 58) | 42.9 ± 16.2 | (10.0, 4.3–76.5) | |
| Growth pattern | | | | |
| Expanding | ($n$ = 47) | 33.7 ± 16.1 | (29.5, 7.7–76.0) | NS |
| Infiltrative | ($n$ = 61) | 45.8 ± 14.5 | (46.0, 15.1–76.5) | |
| Lymph node metastasis | | | | |
| Negative | ($n$ = 59) | 31.5 ± 12.6 | (29.1, 7.7–67.0) | <0.01 |
| Positive | ($n$ = 49) | 49.8 ± 14.0 | (48.5, 10.0–76.5) | |

between MVC, PCNA LI and overall survival, tumours were separated on the basis of the mean values for MVC and PCNA LI (MVC, 16; PCNA LI, 42). There was a significant survival advantage in patients with low-MVC (less than 16) tumours compared with those with high-MVC (16 or greater) tumours (Figure 3a). The 5 year survival rate was 84.6% (55/65) in the patients with low-MVC tumours, but only 55.8% (24/43) in the patients with high-MVC tumours. The 5 year survival rate in the patients with low-PCNA (less than 42) tumours was 86.9% (53/61), which was significantly higher than in the patients with high-PCNA (42 or greater) tumours, 55.8% (26/47) (Figure 3b).

The effects of variables presumably associated with prognosis were studied by Cox's proportional hazard model. As a result, only MVC and PCNA LI emerged as independent prognostic factors (Table III). Of these parameters, PCNA LI was the most important factor for predicting overall survival, followed by MVC.

### *The relationship between the mode of recurrence, MVC and PCNA LI*

With regard to the site of the first relapse, 11 patients had hepatic recurrences, 13 had peritoneal recurrences and five had other sites of recurrence. The relationship between MVC, PCNA LI and recurrence is shown in Table IV. Of the patients with high-MVC tumours, 11 (57.9%) had hepatic recurrence and seven (36.8%) had peritoneal recurrence. In contrast, in patients with low-MVC tumours, six (60%) had peritoneal recurrence and none had hepatic recurrence. The frequency of hepatic recurrence was significantly ($P<0.01$) higher in patients wtih high-MVC tumours than in those with low-MVC tumours. In contrast, there was no significant correlation between PCNA LI and the site of recurrence.

The rates of recurrence subdivided according to MVC and PCNA LI are shown in Figure 4. The rate of recurrence was only 2.4% (1/42) in patients with low-MVC and low-PCNA

**Figure 3** Survival curves of patients with gastric carcinoma subdivided according to MVC (**a**) or PCNA LI (**b**).

LI tumours, wich was significantly ($P<0.01$) lower than in other patients. The highest rate of recurrence was observed in patients with high-MVC and high-PCNA LI tumours. In this group, hepatic recurrences were observed in six (25.0%) patients and peritoneal metastases were observed in five (20.8%) patients.

We determined which factors were related to the site of recurrence by logistic regression analysis. Only MVC was associated significantly with hepatic metastasis. However, PCNA LI was not associated with any type of the recurrence (Table V).

**Table III** Risk factors affecting overall survival determined by Cox's proportional hazard model

| *Variable* | *Hazard ratio* | *95% confidence interval* | *P-value* |
|---|---|---|---|
| Serosal invasion<br>Negative<br>Positive | 1.04 | 0.80–2.71 | 0.076 |
| Histological type<br>Differentiated<br>Undifferentiated | 0.85 | 0.43–1.66 | 0.630 |
| Growth pattern<br>Expanding<br>Infiltrative | 0.96 | 0.75–1.77 | 0.125 |
| Lymph node metastasis<br>Negative<br>Positive | 1.34 | 0.66–3.41 | 0.061 |
| Microvessel count<br>⩾16<br><16 | 2.15 | 1.07–4.26 | 0.030 |
| PCNA labelling index<br>⩾42<br><42 | 3.07 | 1.84–6.17 | 0.021 |

**Table IV** Recurrent cases after curative resection

| | *Rate of recurrence* | *Location of recurrence* | |
|---|---|---|---|
| *MVC* | | | |
| ⩾16 (*n* = 43) | 44.2 (19/43) ]* | Liver | 57.9 (11/19) ]* |
| | | Peritoneum | 36.8 (7/19) |
| | | Other | 5.3 (1/19) |
| <16 (*n* = 65) | 15.4 (10/65) | Liver | 0 (0/10) |
| | | Peritoneum | 60.0 (6/10) |
| | | Other | 40.0 (4/10) |
| *PCNA LI* | | | |
| ⩾42 (*n* = 47) | 44.7 (21.47) ]* | Liver | 28.6 (6/21) |
| | | Peritoneum | 47.6 (10/21) |
| | | Other | 23.8 (5/21) |
| <42 (*n* = 61) | 13.1 ( 8/61) | Liver | 62.5 (5/8) |
| | | Peritoneum | 37.5 (3/8) |
| | | Other | 0 (0/8) |

*Statistical differences were calculated as $P < 0.01$ by the chi-square test.

### Discussion

It is now well established that vascularity and proliferative activity play key roles in tumour growth, invasiveness and metastasis (Van Dierendonck *et al.*, 1989; Folkman, 1992; Weidner *et al.*, 1993). Recently, it has been suggested that the degree of tumour angiogenesis is related to clinical outcome, suggesting that angiogenic properties correlate with tumour aggressiveness (Weidner *et al.*, 1992; Gasparini *et al.*, 1994). In our study, there were no significant associations between histological type, depth of invasion and MVC. However, MVC was significantly higher in patients with lymph node metastases than in those without such metastases. Weider *et al.* (1992) and Bosari *et al.* (1992) have also reported a significant correlation between microvessel density and the presence of metastatic disease in invasive breast cancer.

In addition, as measurement of cellular proliferative activity has been suggested to be effective in judging the malignant potential of various carcinomas (Van Dierendonck *et al.*, 1989), cell proliferation has been measured by means of DNA ploidy analysis (Sowa *et al.*, 1988), bromodeoxyuridine (Wilson *et al.*, 1986) or Ki-67 (Bouzubar *et al.*, 1989) immunostaining. PCNA is an auxiliary protein of DNA polymerase $\delta$ which plays a major role in DNA syn-

**Figure 4** Correlation between the rate of recurrence, MVC and PCNA LI. ▭, liver; ■, peritoneum, ▨, other.

**Table V** Risk factors affecting recurrence by logistic regression analysis

| | *Location of recurrence* | | | | | |
|---|---|---|---|---|---|---|
| | *Liver* | | *Peritoneum* | | *Other* | |
| *Variable* | *Relative risk* | *P-value* | *Relative risk* | *P-value* | *Relative risk* | *P-value* |
| Serosal invasion<br>Negative<br>Positive | 1.49 | 0.138 | 1.93 | 0.044 | 0.06 | 0.953 |
| Histological type<br>Differentiated<br>Undifferentiated | 1.70 | 0.094 | 0.16 | 0.867 | 0.28 | 0.779 |
| Growth pattern<br>Expanding<br>Infiltrative | 0.47 | 0.634 | 1.30 | 0.196 | 0.63 | 0.530 |
| Lymph node metastasis<br>Negative<br>Positive | 0.86 | 0.391 | 0.21 | 0.833 | 1.17 | 0.244 |
| Microvessel count<br>⩾16<br><16 | 2.40 | 0.018 | 1.45 | 0.152 | 1.40 | 0.163 |
| PCNA labelling index<br>⩾42<br><42 | 1.06 | 0.290 | 1.69 | 0.095 | 1.38 | 1.168 |

thesis and is thought to be expressed in the nuclei particularly in late $G_1$ and S-phases (Garcia *et al.*, 1989; Landberg *et al.*, 1990). In this study, we used PCNA LI as a marker for cell proliferation. As a result, we found that the higher grade of PCNA expression was observed in patients with lymph node metastasis or serosal invasion. Yonemura *et al.* (1993) reported higher PCNA expression in patients with tumours of diameter 6 cm or more and with lymph node metastasis.

With regard to prognosis, our study demonstrated significantly poorer prognosis in patients with high-MVC tumours or high-PCNA LI tumours, and both MVC and PCNA LI were independent significant prognostic factors. Weidner *et al.* (1992) have shown that both relapse-free and overall survival rates decrease with increasing MVC. Toi *et al.* (1993) have also reported that MVC is an independent prognostic factor in patients with breast cancer. Similarly, Yonemura *et al.* (1993) have reported that, among 120 patients with gastric cancer, those with a high PCNA LI (40% or greater) had a significantly poorer prognosis than those with a low PCNA LI (less than 40%). Furthermore, with regard to sites of recurrence, an increased MVC was significantly associated with hepatic recurrence. These results suggest that both MVC and PCNA LI are prognostic indicators and that MVC is an effective predictor of hepatic recurrences in patients with gastric carcinoma.

Tumour cells are rarely shed into the circulation before the primary tumour is vascularised (Folkman, 1990). It has been shown that greater numbers of tumour vessels increase the opportunity for tumour cells to enter the circulation (Liotta *et al.*, 1976). Moreover, newly formed capillaries have fragmented basement membranes and are leaky compared with mature vessels, making them penetrable by tumour cells (Nagyl *et al.*, 1988). Therefore, in the high-MVC tumours, the metastatic process may be enhanced by the leaky nature of newly formed blood vessels, which facilitates vascular invasion. Our results confirm the association between high vessel count in gastric carcinoma and the risk of hepatic recurrence.

Various adjuvant therapies have been given to patients with advanced gastric cancer to prevent recurrence after resection. However, 50% of patients with advanced gastric cancer survive after curative resection without post-operative therapy (Miwa, 1984). Therefore, patients who need adjuvant therapies should be selected by some indicators reflecting the probability of recurrence. Thus, MVC and PCNA LI in resected specimens may be suitable methods of identifying patients who need additional therapy post-operatively.

Recently, TNP-470, an analogue of fumagillin derived from *Aspergillus fumigatus*, has been shown to inhibit angiogenesis and the growth of some tumours (Ingber *et al.*, 1990; Yamaoka *et al.*, 1993). Such agents may prove to be valuable anti-tumour chemotherapeutic agents, especially in patients with high-MVC tumours.

In summary, this retrospective study demonstrates that both MVC and PCNA LI may be good prognostic indicators and that MVC may be useful in predicting the hepatic recurrence in patients with gastric carcinoma. If these findings are confirmed in larger studies, it will be possible to add MVC and PCNA LI to other prognostic factors to identify patients at high risk of recurrence and to guide decisions on additional therapy after surgery.

**Abbreviations**: F-VIII RA, factor VIII-related antigen; PCNA, proliferating cell nuclear antigen.

### References

BOSARI S, LEE AKC, DELELLIS RA, WILEY BD, HEATLEY GJ AND SILVERMAN ML. (1992). Microvessel quantitation and prognosis in invasive breast carcinoma. *Hum. Pathol.*, **23**, 755–761.

BOUZUBAR N, WALKER KJ, GRIFFITHS K, ELLIS IO, ELSTON CW, ROBERTSON JF, BLAMEY RW AND NICHOLSON RJ. (1989). Ki-67 immunostaining in primary breast cancer: pathological and clinical associations. *Br. J. Cancer*, **59**, 943–947.

COX DR. (1972). Regression models and life tables. *J.R. Stat. Soc. B.*, **34**, 187–220.

FOLKMAN J. (1990). What is the evidence that tumours are angiogenesis dependent? *J. Natl Cancer Inst.*, **82**, 4–6.

FOLKMAN J. (1992). The role of angiogenesis in tumour growth. *Semin. Cancer Biol.*, **3**, 65–71.

GARCIA RL, COLTRERA MD AND GOWN AM. (1989). Analysis of proliferative grade using anti PCNA/cyclin in fixed, embedded tissues. *Am. J. Pathol.*, **134**, 733–739.

GASPARINI G, WEIDNER N, BEVILACQUA P, MALUTA S, PALMA PD, CAFFO O, BARBARESCHI M, BORACCHI P, MARUBINI E AND POZZA F. (1994). Tumour microvessel density, p53 expression, tumour size, and peritumoural lymphatic vessel invasion are relevant prognostic marker in node-negative breast carcinoma. *J. Clin. Oncol.*, **12**, 454–466.

INGBER D, FUJITA T, KISHIMOTO S, SUDO K, KANAMARU T, BREM H AND FOLKMAN J. (1990). Synthetic analogues of fumagillin that inhibit angiogenesis and suppress tumour growth. *Nature*, **384**, 555–557.

JAPANESE RESEARCH SOCIETY FOR GASTRIC CANCER. (1981). The general rules for gastric cancer study. *Jpn J. Surg.*, **11**, 127–139.

LANDBERG G, TAN EM AND ROOS G. (1990). Flow cytometric multiparameter analysis of proliferating cell nuclear antigen/cyclin and Ki-67 antigen. *Exp. Cell Res.*, **187**, 111–118.

LIOTTA LA, KLEINERMAN J AND SAIDEL G. (1976). The significance of hematogenous tumour cell clumps in the metastatic process. *Cancer Res.*, **36**, 889–894.

MIWA K. (1984). Evaluation of TNM classification of stomach cancer and proposal for its rational stage-grouping. *Jpn J. Clin. Oncol.*, **14**, 385–410.

NAGYL JA, BROWN LF, SENGER DR, LANIR N, VAN DE WATER L, DVORAK AM AND DVORAK HF. (1988). Pathogenesis of tumour stroma generation: a critical role for leaky blood vessels and fibrin deposition. *Biochim. Biophys. Acta*, **948**, 305–326.

SOWA M, YOSHINO H, KATO Y, NISHIMURA M, KAMINO K AND UMEYAMA K. (1988). An analysis of the DNA ploidy patterns of gastric cancer. *Cancer*, **62**, 1325–1330.

TOI M, KASHITANI J AND TOMONAGA T. (1993). Tumour angiogenesis is an independent prognostic indicator in primary breast carcinoma. *Int. J. Cancer*, **55**, 371–374.

VAN DIERENDONCK JH, KEIJZER R, VAN DE VELDE CJH AND CORNELISSE CJ. (1989). Nuclear distribution of the Ki-67 antigen during the cell cycle – comparison with growth fraction in human breast cancer cells. *Cancer Res.*, **49**, 2999–3006.

WEIDNER N, FOLKMAN J, POZZA F, BEVILACQUA P, ALLRED EN, MOORE DH, MELI S AND GASPARINI G. (1992). Tumour angiogenesis: a new significant and independent prognostic indicator in early-stage breast carcinoma. *J. Natl Cancer Inst.*, **84**, 1875–1887.

WEIDNER N, CARROLL PR, FLAX J, BLUMENFELD W AND FOLKMAN J. (1993). Tumour angiogenesis correlates with metastasis in invasive prostate carcinoma. *Am. J. Pathol.*, **143**, 401–409.

WILSON GD, MACNALLY NJ, DUNPHY E, KRACHER H AND PRAFGNA R. (1986). The labeling index of human and mouse tumours assessed by bromodeoxyuridine staining *in vitro* and *in vivo* flow cytometry. *Cytometry*, **6**, 641–647.

YAMAOKA M, YAMAMOTO T, MAKAKI T, IKEYAMA S, SUDO K AND FUJITA T. (1993). Inhibition of tumour growth and metastasis of rodent tumours by the angiogenesis inhibitor *o*-(chloroacetyl-carbamoyl) fumagillol (TNP-470; AGM-1470). *Cancer Res.*, **53**, 4262–4267.

YONEMURA Y, KIMURA K, FUSHIDA Y, TUGAWA K, NAKAI Y, KAJI M, FONSECA L, YAMAGUCHI A AND MIYAZAKI I. (1993). Analysis of proliferative activity using anti-proliferative cell nuclear antigen in gastric cancer tissue specimens obtained by endoscopic biopsy. *Cancer*, **71**, 2448–2453.


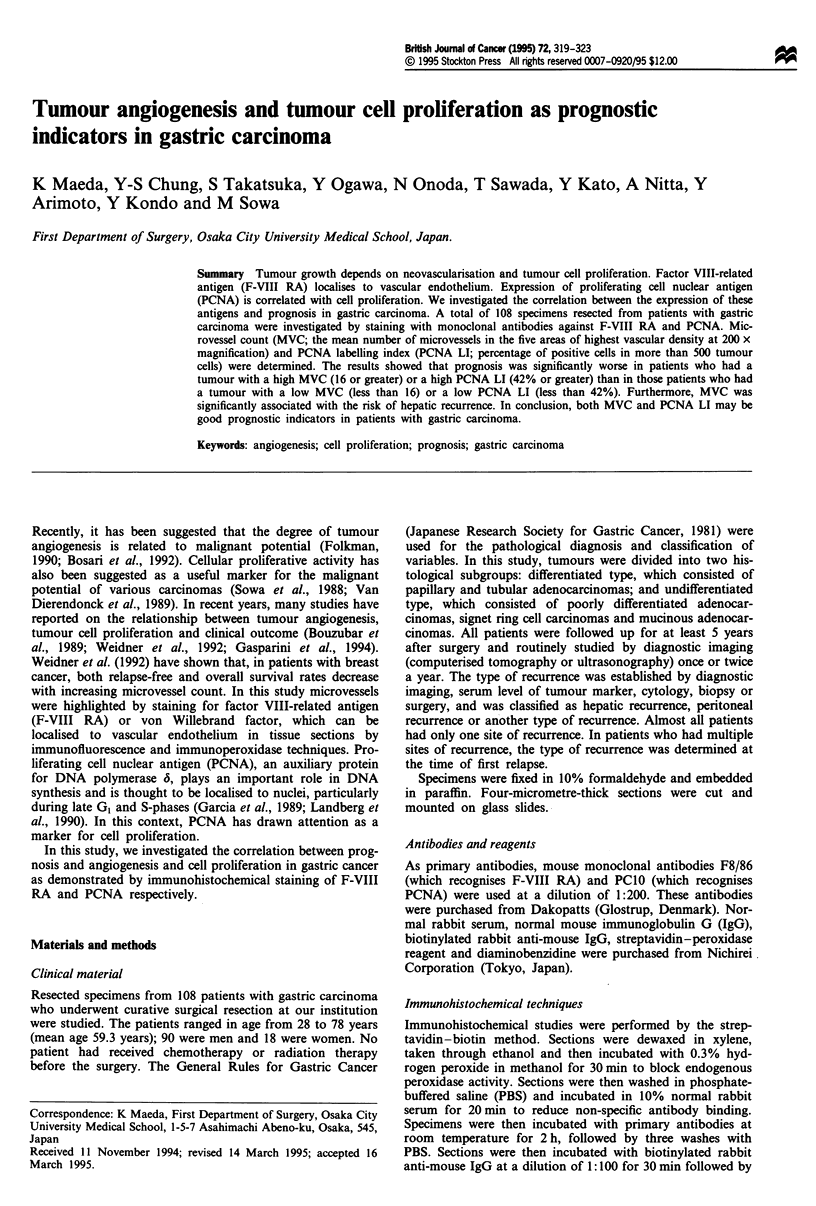

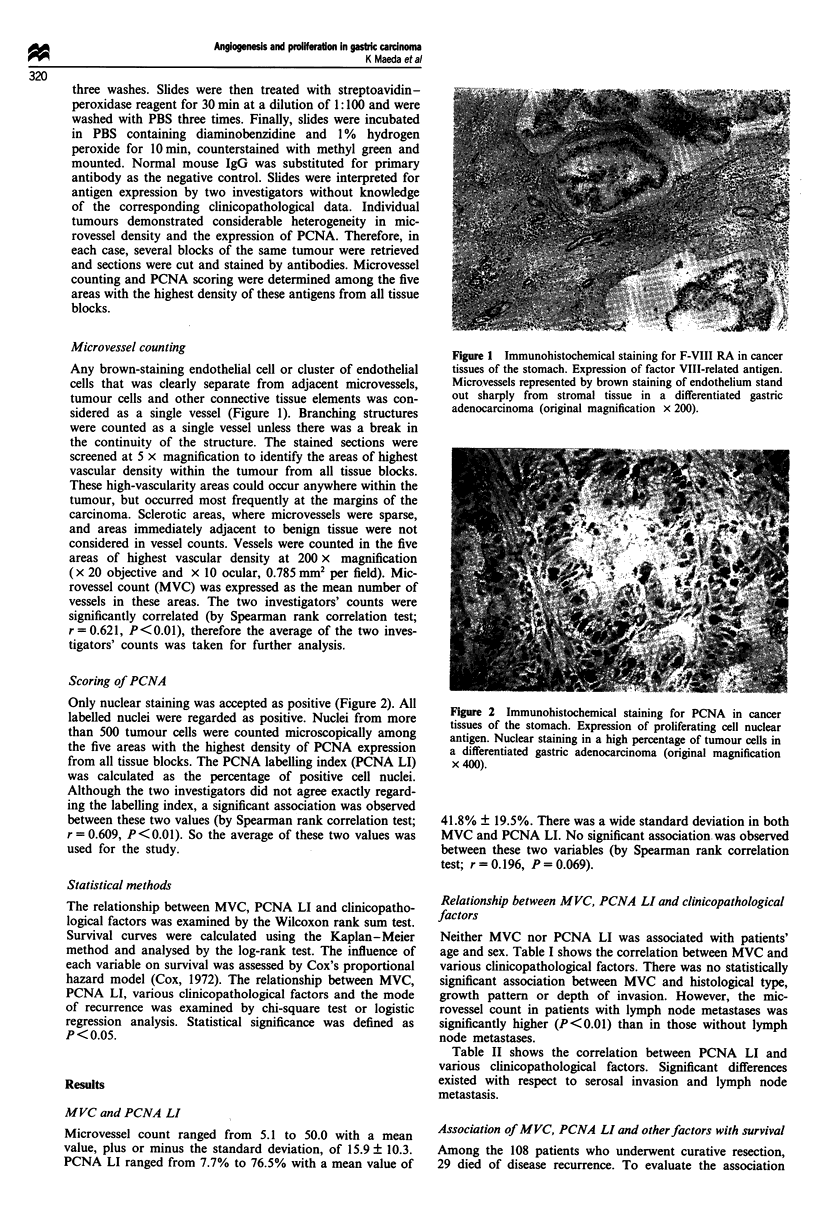

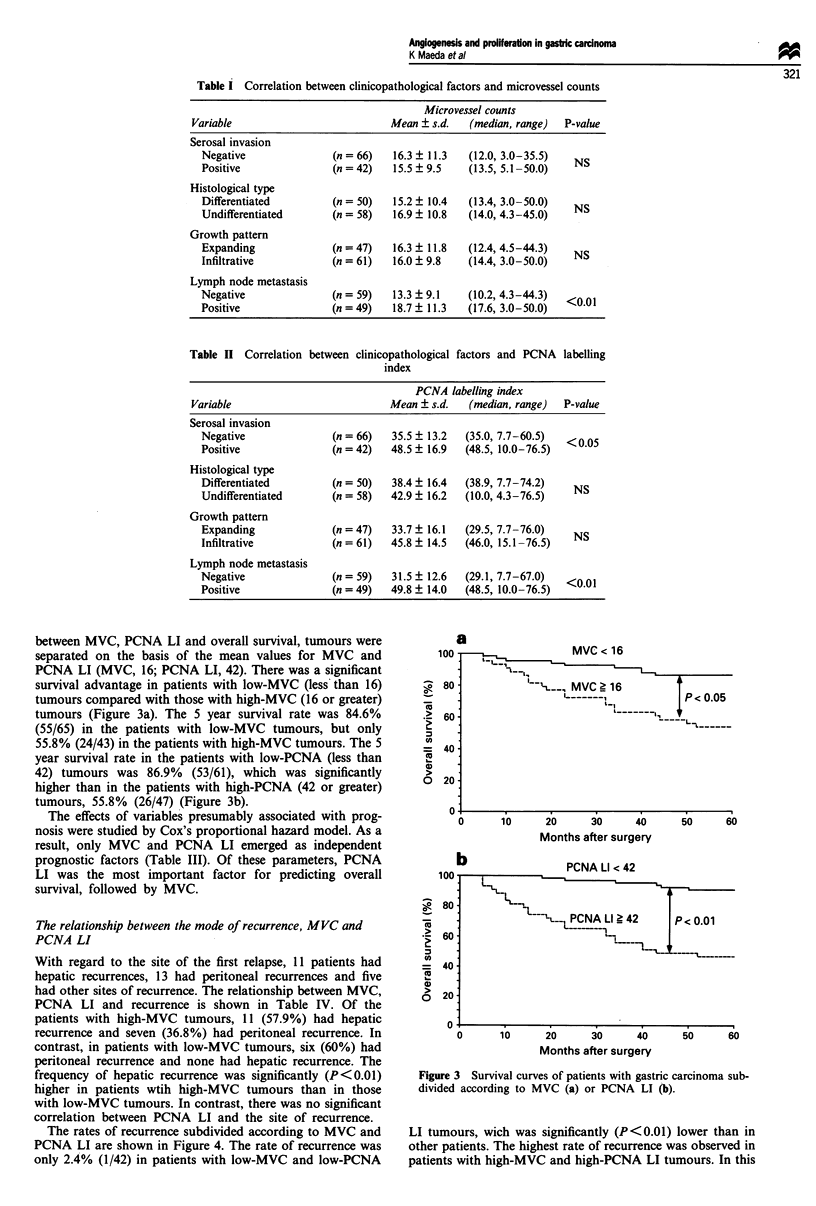

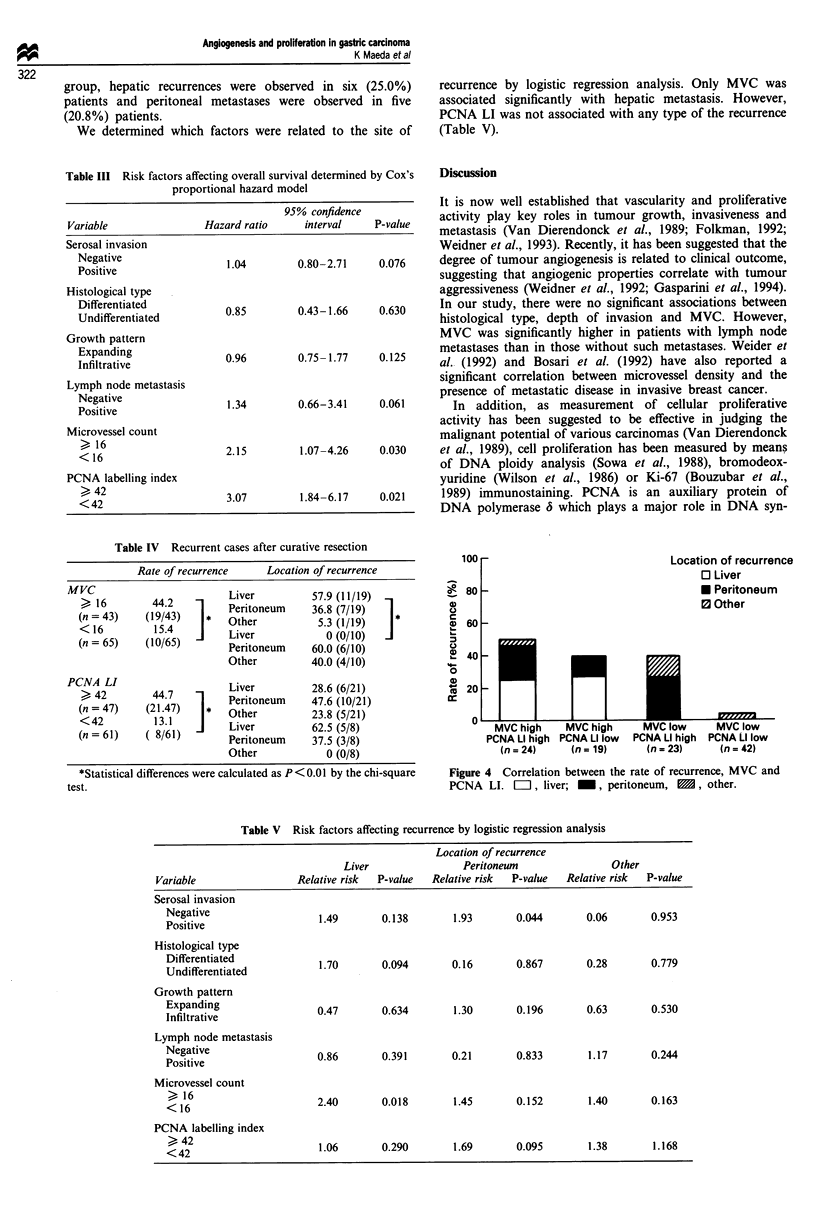

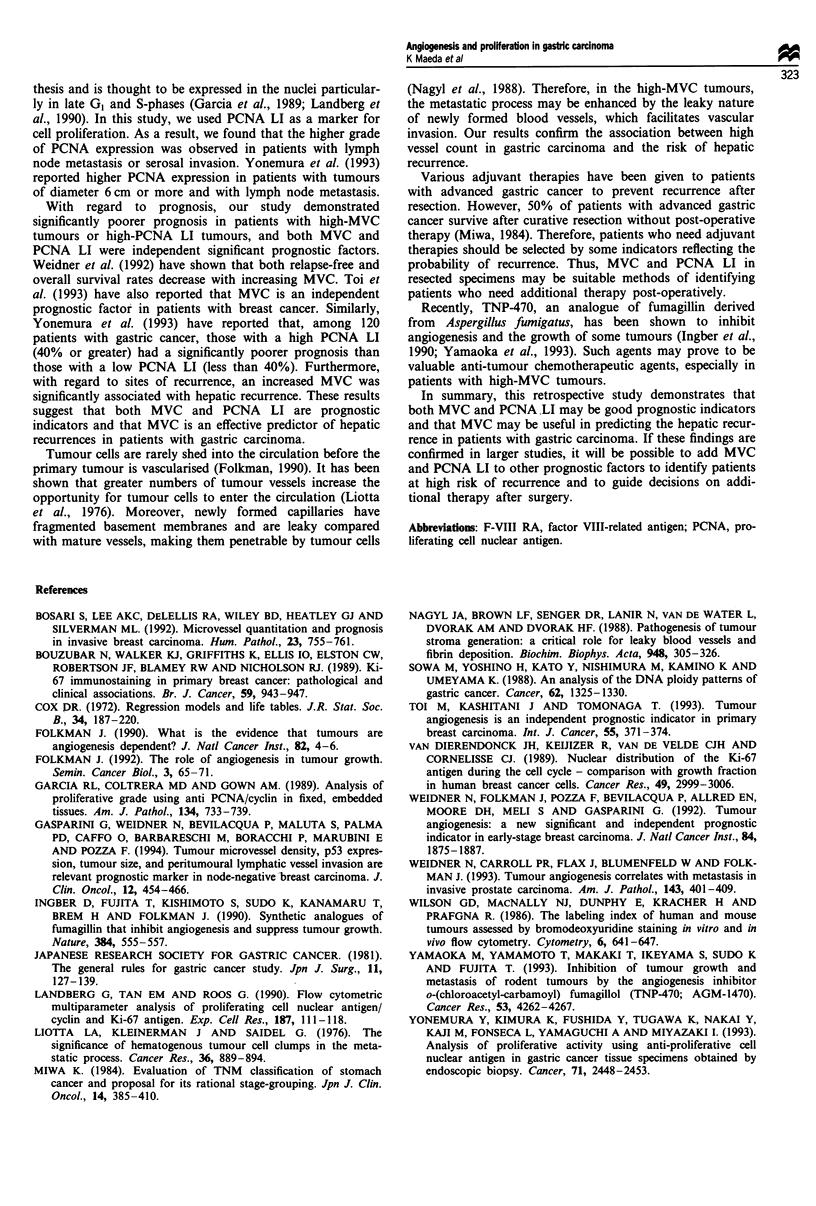


## References

[OCR_00645] Bosari S., Lee A. K., DeLellis R. A., Wiley B. D., Heatley G. J., Silverman M. L. (1992). Microvessel quantitation and prognosis in invasive breast carcinoma.. Hum Pathol.

[OCR_00647] Bouzubar N., Walker K. J., Griffiths K., Ellis I. O., Elston C. W., Robertson J. F., Blamey R. W., Nicholson R. I. (1989). Ki67 immunostaining in primary breast cancer: pathological and clinical associations.. Br J Cancer.

[OCR_00663] Folkman J. (1992). The role of angiogenesis in tumor growth.. Semin Cancer Biol.

[OCR_00665] Garcia R. L., Coltrera M. D., Gown A. M. (1989). Analysis of proliferative grade using anti-PCNA/cyclin monoclonal antibodies in fixed, embedded tissues. Comparison with flow cytometric analysis.. Am J Pathol.

[OCR_00670] Gasparini G., Weidner N., Bevilacqua P., Maluta S., Dalla Palma P., Caffo O., Barbareschi M., Boracchi P., Marubini E., Pozza F. (1994). Tumor microvessel density, p53 expression, tumor size, and peritumoral lymphatic vessel invasion are relevant prognostic markers in node-negative breast carcinoma.. J Clin Oncol.

[OCR_00678] Ingber D., Fujita T., Kishimoto S., Sudo K., Kanamaru T., Brem H., Folkman J. (1990). Synthetic analogues of fumagillin that inhibit angiogenesis and suppress tumour growth.. Nature.

[OCR_00691] Landberg G., Tan E. M., Roos G. (1990). Flow cytometric multiparameter analysis of proliferating cell nuclear antigen/cyclin and Ki-67 antigen: a new view of the cell cycle.. Exp Cell Res.

[OCR_00694] Liotta L. A., Saidel M. G., Kleinerman J. (1976). The significance of hematogenous tumor cell clumps in the metastatic process.. Cancer Res.

[OCR_00699] Miwa K. (1984). Evaluation of the TNM classification of stomach cancer and proposal for its rational stage-grouping.. Jpn J Clin Oncol.

[OCR_00704] Nagy J. A., Brown L. F., Senger D. R., Lanir N., Van de Water L., Dvorak A. M., Dvorak H. F. (1989). Pathogenesis of tumor stroma generation: a critical role for leaky blood vessels and fibrin deposition.. Biochim Biophys Acta.

[OCR_00710] Sowa M., Yoshino H., Kato Y., Nishimura M., Kamino K., Umeyama K. (1988). An analysis of the DNA ploidy patterns of gastric cancer.. Cancer.

[OCR_00717] Toi M., Kashitani J., Tominaga T. (1993). Tumor angiogenesis is an independent prognostic indicator in primary breast carcinoma.. Int J Cancer.

[OCR_00735] Weidner N., Carroll P. R., Flax J., Blumenfeld W., Folkman J. (1993). Tumor angiogenesis correlates with metastasis in invasive prostate carcinoma.. Am J Pathol.

[OCR_00728] Weidner N., Folkman J., Pozza F., Bevilacqua P., Allred E. N., Moore D. H., Meli S., Gasparini G. (1992). Tumor angiogenesis: a new significant and independent prognostic indicator in early-stage breast carcinoma.. J Natl Cancer Inst.

[OCR_00740] Wilson G. D., McNally N. J., Dunphy E., Kärcher H., Pfragner R. (1985). The labelling index of human and mouse tumours assessed by bromodeoxyuridine staining in vitro and in vivo and flow cytometry.. Cytometry.

[OCR_00744] Yamaoka M., Yamamoto T., Masaki T., Ikeyama S., Sudo K., Fujita T. (1993). Inhibition of tumor growth and metastasis of rodent tumors by the angiogenesis inhibitor O-(chloroacetyl-carbamoyl)fumagillol (TNP-470; AGM-1470).. Cancer Res.

[OCR_00751] Yonemura Y., Kimura H., Fushida S., Tugawa K., Nakai Y., Kaji M., Fonseca L., Yamaguchi A., Miyazaki I. (1993). Analysis of proliferative activity using anti-proliferating cell nuclear antigen antibody in gastric cancer tissue specimens obtained by endoscopic biopsy.. Cancer.

[OCR_00722] van Dierendonck J. H., Keijzer R., van de Velde C. J., Cornelisse C. J. (1989). Nuclear distribution of the Ki-67 antigen during the cell cycle: comparison with growth fraction in human breast cancer cells.. Cancer Res.

